# Ecological parameter reductions, environmental regimes, and characteristic process diagram of carbon dioxide fluxes in coastal salt marshes

**DOI:** 10.1038/s41598-020-72066-8

**Published:** 2020-09-25

**Authors:** Khandker S. Ishtiaq, Omar I. Abdul-Aziz

**Affiliations:** West Virginia University, P.O. Box 6103, Morgantown, WV 26506-6103 USA

**Keywords:** Carbon cycle, Carbon cycle

## Abstract

We investigated the ecological parameter reductions (termed “similitudes”) and characteristic patterns of the net uptake fluxes of carbon dioxide (CO_2_) in coastal salt marshes using dimensional analysis method from fluid mechanics and hydraulic engineering. Data collected during May–October, 2013 from four salt marshes in Waquoit Bay and adjacent estuary, Massachusetts, USA were utilized to evaluate the theoretically-derived dimensionless flux and various ecological driver numbers. Two meaningful dimensionless groups were discovered as the light use efficiency number (LUE = CO_2_ normalized by photosynthetically active radiation) and the biogeochemical number (combination of soil temperature, porewater salinity, and atmospheric pressure). A semi-logarithmic plot of the dimensionless numbers indicated the emergence of a characteristic diagram represented by three distinct LUE regimes (high, transitional, and low). The high regime corresponded to the most favorable (high temperature and low salinity) condition for CO_2_ uptake, whereas the low regime represented an unfavorable condition (low temperature and high salinity). The analysis identified two environmental thresholds (soil temperature ~ 17 °C and salinity ~ 30 ppt), which dictated the regime transitions of CO_2_ uptake. The process diagram and critical thresholds provide important insights into the CO_2_ uptake potential of coastal wetlands in response to changes in key environmental drivers.

## Introduction

Coastal wetlands are among the most potent carbon sinks on earth, providing valuable ecosystem services such as global warming mitigation, storm protection, erosion control, and carbon sequestration^1, 2^. Understanding of the wetland carbon dioxide (CO_2_) exchange processes is pivotal to the ecological stability because of the vulnerability of wetland ecosystems to global environmental change^3, 4^. The magnitude and variability of CO_2_ in coastal wetlands depend on their complex nonlinear interplay with the major environmental drivers (e.g., light, temperature, salinity, pressure, and scales of measurements in time and space)^5–7^. However, it remains unexplored whether the primary drivers of wetland CO_2_ fluxes can be grouped into a reduced set of emergent generalizable entities that represent the coastal wetland productivity potentials in response to a changing environment. Therefore, a fundamental scientific question is whether we can identify ecological parametric reductions, known as “similitudes” in the domain of fluid mechanics and hydraulic engineering, describing environmental regimes of salt marsh CO_2_ uptake. Do the salt marsh CO_2_ fluxes represent any characteristic process path (e.g., diagram) with the covarying environmental drivers? Are there any critical environmental thresholds, indicating shifts in the process regimes of CO_2_ fluxes? Answers to these scientific questions using similitude and dimensional analysis from fluid mechanics and hydraulic engineering may offer new insights into the underlying mechanistic principles and into the development of generalized predictive models of salt marsh CO_2_ fluxes in space and time.

Wetlands are, in general, net sink of CO_2_ during daytime due to photosynthesis and source during nighttime because of respiration^8^. There are multitudes of primary process drivers of salt marsh CO_2_ exchanges with the atmosphere. Light is the main driver of net CO_2_ uptake, whereas temperature mediates the photosynthesis process^9^. The temperature control on CO_2_ uptake is intertwined with the activity of the primary photosynthesis enzyme, RuBisCO—which has a positive linkage with ambient temperature^10^. In contrast, salinity significantly reduces the CO_2_ uptake rates and overall productivity in tidal wetlands by influencing the plants’ physiological responses to available light^11, 12^. Usually, salt marsh plants are resilient to high salinity; however, previous studies reported a considerable negative impact of salinity on the productivity of the salt marshes^13, 14^. A high saline condition favors the accumulation of toxic hydrogen sulfide in anaerobic wetland soils—which adversely influences the salt marsh productivity^15^. Furthermore, high salinity reduces the nutrient uptake (e.g., nitrogen and phosphorous) of the marshes, and thereby decreases the productivity^16^.

Typically, salt marsh CO_2_ uptake rates are higher during the high tides than the low tides, because salt accumulation during the low tides negatively impacts the productivity^17^. Abdul-Aziz et al.^7^ carried a comprehensive study to determine the major drivers of daytime CO_2_ uptake in Waquoit Bay and adjacent estuaries, MA, USA using a systematic data analytics and empirical modeling approach. The study reported a substantially higher control and linkage of photosynthetically active radiation, soil temperature, and porewater salinity on instantaneous CO_2_ uptake fluxes than that of pH, well water level, and soil moisture. Further, Abdul-Aziz et al.^7^ did not find any significant control of nitrogen loading gradient (5–126 kg/ha/year) on the CO_2_ uptake fluxes across the study marshes; however, the authors reported significantly higher CO_2_ uptake fluxes during high tides than the low tides.

A crucial advantage of identifying similitudes and dimensionless process groups is that information and underlying relationships and patterns can be generalized by utilizing data from different sites representing different gradients of response and predictor variables. In general, similitude represents the parametric reduction of a physical system by developing independent dimensionless groups or pi (Π) numbers, which functionally defines the mechanistic process of the system^18^. As the Π numbers represent different factors pertinent to the process, the fundamental description of the system defined by the associated process variables remains unchanged. Dimensional analysis using Buckingham pi-theorem is a useful method to decrease dimensions in data, reduce the number of parameters, and achieve similitude. The method is particularly useful in systems where simple governing equations cannot be used to represent the primary processes^18, 19^.

Classical examples of similitudes and dimensional analysis that led to the development of generalized process diagrams and scaling relationships include “Moody diagram” in fluid mechanics and “Shields diagram” in sediment transport^20, 21^. Here, the term “process diagram” refers to a graphical representation of different process states that a system follows in transition from one balanced condition to another. In “Moody Diagram”, seven original process variables of pipe flow were reduced to three process-based dimensionless groups using dimensional analysis, representing pipe friction and roughness across laminar, transitional, and turbulent flow regimes. Similarly, six process variables in rivers were used to formulate two meaningful dimensionless groups in “Shields diagram”, indicating different states of sediment transport across smooth, transitional, and rough flow regimes. Many studies in ecology, environmental sciences, and engineering utilized the concept of parametric reductions and dimensional analysis for process understanding and modeling^22–30^. West et al.^22^ employed parametric reductions and scaling to derive a single universal curve to describe the growth rate of diverse species. Warnaars et al.^24^ applied dimensional analysis for parametric reductions in stream biogeochemistry, and developed useful scaling relationships between dimensionless biotic and abiotic factors in various streams across North America.

This study tests a fundamental hypothesis that the net CO_2_ uptake fluxes in coastal salt marshes follow emergent ecological parameter reductions (i.e., similitudes) and distinct environmental regimes. The hypothesis was evaluated by formulating meaningful dimensionless numbers and defining different process regimes of salt marsh CO_2_ uptake fluxes, leading to a characteristics process diagram. The field data collected from four salt marshes on the southern shore of Cape Cod, MA, USA were used to examine the hypothesis.

## Materials and methods

### Study wetlands and datasets

**Figure 1:**
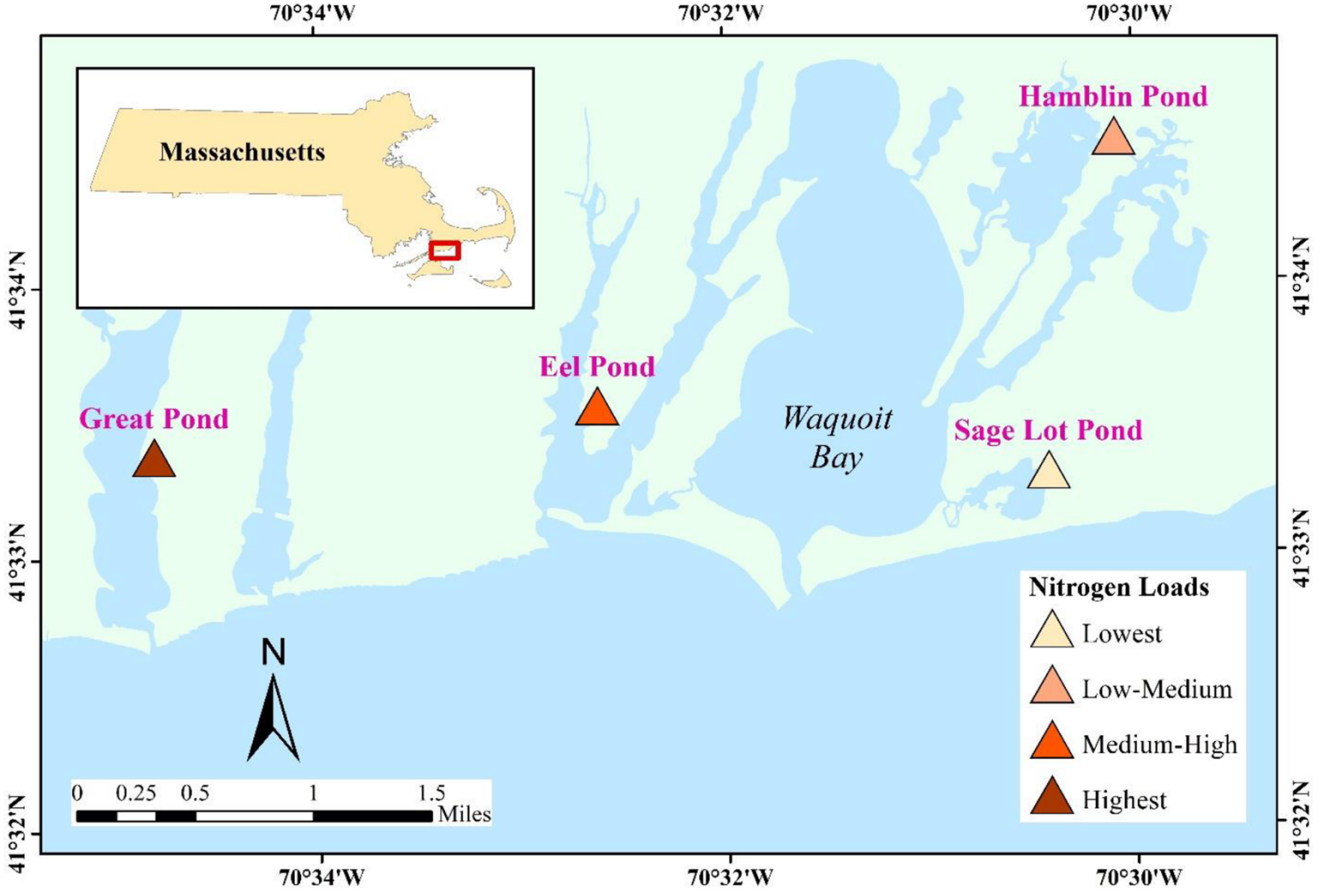
Locations of the case study salt marshes along the southern shore of Cape Cod in the Waquoit Bay and adjacent estuaries, MA. Nitrogen loading rates of the Sage Lot Pond, Hamblin Pond, Eel Pond, and Great Pond were 5, 29, 63, and 126 kg/ha/year, respectively.

Four salt marshes located in Waquoit Bay and adjacent estuaries at Cape Cod, MA, USA were used as the case study sites: (1) Sage Lot Pond (SL), (2) Eel Pond (EP), (3) Great Pond (GP), and (4) Hamblin Pond (HP) (Fig. 1). The marshes represent a moderate gradient in nitrogen loading and a wide range of human population density^31, 32^. On the basis of nitrogen loading influx, SL is in relatively pristine condition (~ 5 kg/ha/year), whereas HP (~ 29 kg/ha/year), EP (~ 63 kg/ha/year), and GP (~ 126 kg/ha/year) represent a medium to high nitrogen loading^31, 33^. The vegetation community of the marshes is mostly dominated by *Spartina alterniflora* (a C_4_ plant) in the low marsh zone.

A comprehensive detail on the collections and processing of gas fluxes and environmental variables for the four salt marshes were presented in Abdul-Aziz et al.^7^. Closed chamber-based measurements of the net ecosystem exchange (NEE) of CO_2_ were made using a cavity ring-down spectrometer (CRDS) gas-analyzer (Model G2301, Picarro, Inc., Santa Clara, CA; frequency: 1 Hz; precision: 0.4 ppm) for different days during the extended growing season (May to October) in 2013 at the low marsh zones of the four salt marshes. The spectrometer analyzer was connected to the transparent, closed acrylic chamber (60 cm × 60 cm × 60 cm) through tubes. We calculated the molar concentrations of CO_2_ in the chamber using the ideal gas law. The instantaneous molar concentrations of CO_2_ were then linearly regressed with time (s). The regression slopes (i.e., rates of changes in CO_2_ concentrations) were normalized by the chamber area (60 cm × 60 cm = 3,600 cm^2^ = 0.36 m^2^) to compute the corresponding fluxes of CO_2_ (i.e., changes in CO_2_ concentrations per unit area and per unit time in μmol/m^2^/s) between the wetland soil and the atmosphere inside the chamber for each sampling period (typically ~ 5 min)^6, 7^. To avoid impacts of any experimental error, a coefficient of determination (R^2^) of 0.90 was set as the minimum threshold for the regression to qualify the computed CO_2_ fluxes as accurate and acceptable for analyses^6, 7^.

The employed enclosed chamber-based technique of measuring CO_2_ fluxes is a widely-used method in the carbon research domain^34–37^. The technique provides an effective way to measure surface-atmospheric gas fluxes. As demonstrated above, the method first involves the calculation of the gradient of molar concentrations of CO_2_ in time, which is then divided by the chamber area to compute the vertical CO_2_ fluxes. Since the measurement chamber is small (e.g., 60 cm × 60 cm × 60 cm for our equipment) and enclosed, the vertical fluxes of CO_2_ between soil and atmosphere (and not the divergence of CO_2_ fluxes) drives the changes in CO_2_ concentrations with time inside the chamber. Therefore, the chamber area-normalized rates of changes in CO_2_ molar concentrations represent the vertical CO_2_ gas fluxes between the soil and atmosphere inside the chamber.

The associated instantaneous environmental variables such as the photosynthetically active radiation (PAR), air temperature (AT), soil temperature (ST), and porewater salinity (SS) were concurrently measured^7^. The corresponding observations of atmospheric pressure (P_a_) were collected from the nearby NOAA National Estuarine Research Reserve System *(*NOAA*-*NERRS*)* monitoring station located at Carriage House, MA^38^. The filtered daytime net uptake fluxes of CO_2_ (NEE_CO2,uptake_) represented the measurements made between 8 a.m. and 4.30 p.m. (Eastern Standard Time, EST), with the corresponding PAR higher than 1.5 µmole/m^2^/s. AT was used to calculate the fluxes of NEE_CO2,uptake_ using the ideal gas law^7^; AT was, therefore, excluded as an environmental driver from further analyses. Instead, soil temperature (ST) was considered to represent the impact of temperature on NEE_CO2,uptake_. The dataset included 137 observational panels from the four study wetlands for 25 sampling days (Table S1, Figure S1 and S2 in Supplemental notes).

### Theoretical formulation of dimensionless numbers through parametric reductions

Dimensional analysis using Buckingham pi ($\Pi$) theorem were applied to formulate wetland ecological similitudes and derive dimensionless functional groups or $\Pi$ numbers^18, 20^. According to the pi theorem, a combination of ${\text{n}}$ dimensional variables would lead to $({\text{n}} - {\text{r}}$) dimensionless Π numbers (${\text{r}} =$ number of relevant fundamental dimensions). NEE_CO2,uptake_, PAR, ST, SS, P_a_, and the time-scale of measurement or estimation (t) were used for the dimensional analysis. PAR, ST, SS were the most dominant drivers of NEE_CO2,uptake,_ as identified in the study of Abdul-Aziz et al.^7^. Furthermore, P_a_ negatively correlates with net photosynthesis as stomatal conductance increases with decreasing pressure^39^. The selected variables for the dimensional analysis included four fundamental dimensions (mass: M; length: L; temperature: K; time: T) (Table 1). Since the variables were in different unitary systems, they were converted to the SI units by using appropriate conversion factors (Table 1). As the temperature dimension (K) was only represented by ST, specific heat of wet soil (c_p_ = 1.48 kJ/kg/K) was further incorporated in the dimensional analysis to normalize ST. Following the pi theorem, a functional relationship ($f$) among the response (NEE_CO2,uptake_) and the potential predictors was expressed as follows:

1 $$f\left( {NEE_{CO2,uptake} , PAR, ST, SS,{ }P_{a} ,{ }c_{p} , t} \right) = 0$$

where the total number of variables, $n = 7$; number of fundamental dimensions, $r = 4$. Therefore, the total number of possible ${\Pi }$ numbers $= {\text{n}} - {\text{r}} = 3$. The functional relation of Eq. (1) was then represented with $\Phi$ in terms of dimensionless numbers as follows:

2 $$\Phi \left( {\Pi _{1} ,\Pi _{2} , \Pi _{3} } \right) = 0$$

**Table 1:** List of variables, units and dimensions used for the dimensional analysis.

Variables	Original units (X)	Converted units (Y)	Units conversation equation	Dimensions
NEE_CO2,uptake_	µmol/m^2^/s	µmol/m^2^/s	Y = X	[ML^−2^ T^−1^]
PAR	µmol/m^2^/s	µmol/m^2^/s	Y = X	[ML^−2^ T^−1^]
ST	°C	K	Y = 273.15 + X	[K]
SS	ppt	g/m^3^	Y = 1,000 * X	[ML^−3^]
P_a_	millibar	g/m/s^2^	Y = 100,000 * X	[ML^−1^ T^−2^]
c_p_ = 1.48	kJ/kg/K	J/g/K	Y = X	[L^2 ^T^−2^ K^−1^]
Time (t)	s	s	Y = X	[T]

NEE_CO2,uptake_, PAR, ST, SS, P_a,_ and c_p_ refer, respectively, to daytime net uptake fluxes of CO_2_, photosynthetically active radiation, soil temperature, porewater salinity, atmospheric pressure, and specific heat of wet soil.

*ppt* refers to parts per thousand.

Based on the pi theorem, four variables ($r = 4$) could be considered as “repeating variables” in each iteration to formulate a dimensionless number by involving any of the remaining variables. Although the repeating variables should include all relevant fundamental dimensions (M, L, K, and T in this study), they should not form a dimensionless number among themselves. For example, considering PAR, ST, SS, and t as the “repeating variables”, the first pi number $\left( {\Pi _{1} } \right)$ was expressed as follows:

3 $$\Pi _{1} = PAR^{a} \cdot ST^{b} \cdot SS^{c} \cdot t^{d} \cdot NEE_{CO2,uptake}$$

where $a$, $b$, $c$, and $d$ were exponents. For $\Pi _{1 }$ to be dimensionless, the following equation was obtained using the principle of dimensional homogeneity (i.e., equal dimensions on both sides):

4 $$M^{0} \cdot L^{0} \cdot T^{0} \cdot K^{0} = \left( {\frac{M}{{L^{2} T}}} \right)^{{\text{a}}} \cdot \left( K \right)^{b} \cdot \left( {\frac{M}{{L^{3} }}} \right)^{c} \cdot \left( T \right)^{d} \cdot \frac{M}{{L^{2} T}}$$

Therefore,

5 $$M^{0} \cdot L^{0} \cdot T^{0} \cdot K^{0} = M^{{{\text{a}} + {\text{c}} + 1}} \cdot L^{{ - 2{\text{a}} - 3c - 2}} \cdot T^{ - a + d - 1} \cdot K^{b}$$

Equating the exponents of M, L, K, and T on both sides, we obtained the following matrix–vector form:

6 $$\left[ {\begin{array}{*{20}c} 1 & 0 & 1 & 0 \\ { - 2} & 0 & { - 3} & 0 \\ { - 1} & 0 & 0 & 1 \\ 0 & 1 & 0 & 0 \\ \end{array} } \right] \left[ {\begin{array}{*{20}c} a \\ b \\ c \\ d \\ \end{array} } \right] = \left[ {\begin{array}{*{20}c} { - 1} \\ 2 \\ 1 \\ 0 \\ \end{array} } \right]$$

The system of linear equations was algebraically solved to compute the exponents as: $a = - 1$, $b = 0$, $c = 0$, and $d = 0$ (see Text S1 in Supplemental notes for detailed algebraic equations and solutions). Therefore, from Eq. (3), we obtained the first pi number as

7 $$\Pi _{1} = \frac{{NEE_{CO2,uptake} }}{PAR}$$

Similarly, the other two ${\Pi }$ numbers were formulated as (see Text S1 in Supplemental notes)

8 $$\Pi_{2} = \frac{{SS \cdot P_{a} }}{{PAR^{2} }}$$

9 $$\Pi_{3} = \frac{{ST \cdot c_{p} \cdot SS^{2} }}{{PAR^{2} }}$$

The pi theorem also allowed the derivation of new $\Pi$ numbers by combining any two (or more) original $\Pi$ numbers through multiplication or division as follows:

10 $$\Pi _{4} =\Pi _{2} \times\Pi _{3} = \frac{{ST \cdot c_{p } \cdot SS^{3} \cdot P_{a} }}{{PAR^{4} }}$$

11 $$\Pi _{5} = \frac{{\Pi _{3} }}{{\Pi _{2} }} = \frac{{ST \cdot c_{p} \cdot SS}}{{P_{a} }}$$

Thus, the functional relationship of Eq. (2) could be represented in any of the following forms:

12 $$\Phi \left( {\Pi _{1} ,\Pi _{4} } \right) = 0$$

13 $$\Phi \left( {\Pi _{1} ,\Pi _{5} } \right) = 0$$

Therefore, dimensional analysis reduced the 7 original variables to 2–3 dimensionless numbers. Recalling the definition of similitude from the physical domain^18, 20^, such parametric reductions for the daytime net uptake fluxes of CO_2_ and the associated environmental drivers were termed as “wetland ecological similitudes” in this research. As apparent, $\Pi _{1}$ represented the dimensionless CO_2_ flux number (i.e., response), whereas $\Pi _{2}$ to $\Pi _{5}$ represented the environmental driver numbers (i.e., predictors).

Various sets of dimensionless numbers were obtained by iteratively changing the “repeating variables” (Table S2; see Text S1 in Supplemental notes for full derivations). However, only the unique ${\Pi }$ numbers were considered for further analysis with empirical data. For example, $\frac{{ SS^{2} \cdot ST \cdot c_{p} }}{{PAR^{2} }}$ (iteration-1 or 4 in Table S2) and $\frac{{SS \cdot \sqrt {ST \cdot c_{p} } }}{PAR}$ (iteration-3) were considered non-unique ${\Pi }$ numbers, because the latter could be obtained as a square root function of the former. Similarly, $\frac{{P_{a} }}{{PAR \cdot \sqrt {ST \cdot c_{p} } }}$ (iteration-3) could be obtained from a square root and inversion of $\frac{{PAR^{2} \cdot ST \cdot c_{p} }}{{P_{a}^{2} }}$ (iteration-2 or 5), and were considered the same number. Based on the pi theorem, the response ${\Pi }$ number (i.e., dimensionless CO_2_ flux number) were expressed as a general function ($\psi$) of all unique dimensionless environmental numbers as follows:

14 $$\frac{{NEE_{CO2,uptake} }}{PAR} = \psi \left[ {\left( {\frac{{SS \cdot P_{a} }}{{PAR^{2} }}} \right),\left( {\frac{{ST \cdot c_{p} \cdot SS^{2} }}{{PAR^{2} }}} \right),\left( {\frac{{ST \cdot c_{p } \cdot SS^{3} \cdot P_{a} }}{{PAR^{4} }}} \right),\left( {\frac{{ST \cdot c_{p} \cdot SS}}{{P_{a} }}} \right),\left( {\frac{{PAR^{2} \cdot ST \cdot c_{p} }}{{P_{a}^{2} }}} \right),\left( {\frac{{SS \cdot P_{a}^{3} }}{{PAR^{4} \cdot ST \cdot c_{p} }}} \right)} \right]$$

### Empirical analysis to determine the linkages among the derived numbers

The multivariate method of principal component analysis (PCA) was applied to the observational dataset from the salt marshes of Waquoit Bay to identify the important environmental driver number(s) that had dominant linkage(s) with the response pi number^40^. PCA can resolve multicollinearity (mutual correlations) among the environmental driver numbers in a multivariate space, identifying the relatively unbiased information on their individual linkages with the response^40, 41^. To incorporate any non-linearity in the data matrix, observed (i.e., calculated) values of all pi numbers were log_10_-transformed, which were further standardized (centralized and scaled) as follows: $Z = \left( {X - \overline{X}} \right)/s_{X}$; $X$ = log_10_-transformed pi number, $\overline{X}$ = mean of $X$, and $s_{X}$ = standard deviation of $X$.

## Results

**Figure 2:**
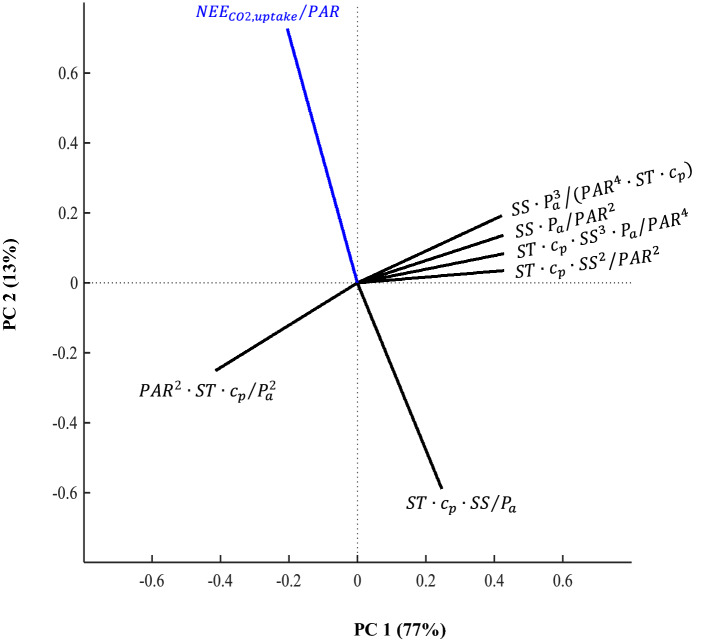
Biplots from principal component (PC) analysis, showing the interrelations, orientations, and groupings of the environmental driver dimensionless numbers with the response dimensionless number $\left( {\frac{{NEE_{CO2,uptake} }}{PAR}} \right)$. NEE_CO2,uptake_, PAR, ST, SS, P_a,_ and c_p_ refer, respectively, to daytime net uptake fluxes of CO_2_, photosynthetically active radiation, soil temperature, porewater salinity, atmospheric pressure, and specific heat of wet soil.

Based on the existing literature, $\frac{{NEE_{CO2,uptake} }}{PAR}$ was termed the “light use efficiency” (LUE) number, which represented salt marsh CO_2_ uptake relative to the available sunlight (PAR)^42–44^. Overall, the first two principal components (PCs) explained 90% of total variance in the dataset when PCA was performed using all unique dimensionless numbers (Eq. 14). The loadings of various dimensionless numbers on the first two PCs were presented in a biplot (Fig. 2), which indicated their interrelations, relative orientations, and groupings with vector lines. The nearly collinear (180°) orientation between LUE and $\frac{{ST \cdot c_{p} \cdot SS}}{{P_{a} }}$ suggested a high linkage between them. Similarly, the approximate collinearity (0° or 180° orientations) of vector lines for the remaining five environmental driver pi numbers indicated their strong interrelationships. However, the nearly orthogonal (90°) orientations of LUE with these other environmental driver numbers indicated their lack of linkages with the CO_2_ flux number. Therefore, $\frac{{ST \cdot c_{p} \cdot SS}}{{P_{a} }}{ }$ was considered as the most important and independent environmental driver number for LUE. $\frac{{ST \cdot SS \cdot c_{p} }}{{P_{a} }}$ was, therefore, termed the “biogeochemical” (BGC) number. Overall, dimensional analysis and PCA together reduced seven original variables to only two dimensionless groups. Based on Eq. (14), the LUE number was then expressed as the sole function of the BGC number as follows:

15 $$\frac{{NEE_{CO2,uptake} }}{PAR} = \psi \left[ {\frac{{ST \cdot SS \cdot c_{p} }}{{P_{a} }}{ }} \right]$$

The derived “ecological similitude” is, therefore, a fundamental contribution in the field of atmosphere-wetland CO_2_ fluxes. The application and usefulness of Eq. (15) in determining the emergent characteristic pattern, process regimes, and underlying relationships were then tested using the collected dataset from the four salt marshes of Cape Cod, MA.

**Figure 3:**
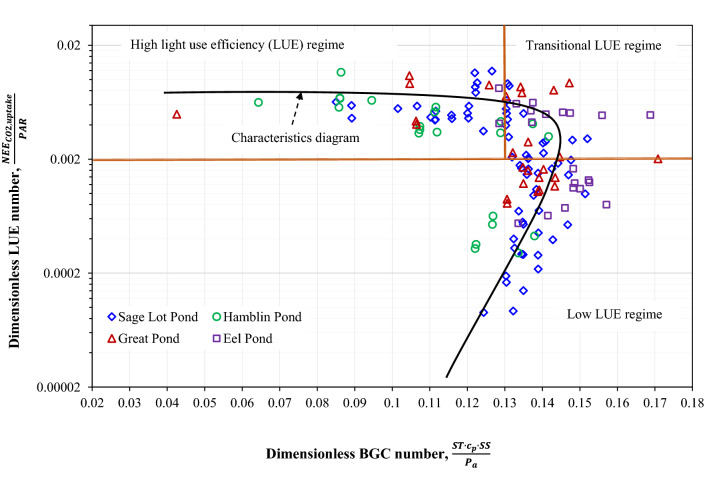
Plot of the dimensionless BGC number with the response dimensionless LUE number, revealing collapse of different variables on the generalized characteristics process diagram for wetland CO_2_ fluxes. LUE refers to the dimensionless light use efficiency number $\left( {\frac{{NEE_{CO2,uptake} }}{PAR}} \right)$ and BGC refers to dimensionless biogeochemical number $\left( {\frac{{ST \cdot c_{p} \cdot SS}}{{P_{a} }}} \right)$. NEE_CO2,uptake_, PAR, ST, SS, c_p_, and P_a_ refer, respectively, to daytime net uptake fluxes of CO_2_, photosynthetically active radiation, soil temperature, porewater salinity, specific heat of wet soil, and atmospheric pressure. The units of NEE_CO2,uptake_ and PAR are in µmol/m^2^/s, ST is in Kelvin, SS is in g/m^3^, c_p_ is in J/g/K, and P_a_ is in g/m/s^2^. The value of constant c_p_ = 1.48 J/g/K.

Semi-logarithmic plot of the dimensionless LUE group as a function of the dimensionless BGC group showed a collapse of observed data from the four salt marshes into a unique dimensionless curve—indicating an emergence of similitude-based characteristic process diagram (Fig. 3). Two distinct regimes were identified from the plot along with a transitional regime that links the two. The zone defined by the BGC ≤ 0.13 and LUE ≥ 0.002 represents the high LUE regime, whereas LUE < 0.002 zone represents the low LUE regime. The transitional LUE regime (BGC > 0.13 and LUE ≥ 0.002) connects the high and low LUE regimes. One-way ANOVA was utilized to test the null hypothesis of no significant difference in LUE between the respective regimes. Based on the ANOVA, the LUE in the high LUE regime was significantly different from the LUE values representing transitional (F_1,103_ = 32.70, *p* value < 0.0001) or low (F_1,72_ = 175.92, *p* value < 0.0001) regime (Table S3). Similarly, LUE values representing the transitional regime differed significantly from the low LUE regime (F_1,93_ = 48.15, *p* value < 0.0001). Overall, the developed curve indicated that CO_2_ uptake relative to light in coastal wetlands was characterized by the corresponding variability in temperature and salinity, and the interactions thereof. The shift from high to transitional regime was determined by the BGC value of 0.13 (Fig. 3). The CO_2_ uptake efficiency had been consistently high for different reasonable combinations of ST and SS until BGC = 0.13 threshold was reached. The LUE, in general, decreased with increasing BGC when the curve crosses the 0.13 limit. However, once the curve had reached the hypothetical tipping point (LUE = 0.0028, BGC = 0.145), LUE dropped sharply with the decrease of BGC values—forming the multivalue-based “loop” shape of the curve.

The region of high LUE regime corresponded to the relatively high temperature (ST ≥ 17 °C), and low to high salinity (10–30 ppt) (Table 2). The associated NEE_CO2,uptake_ and PAR varied within a wide range indicating their high variability, although the range of $\frac{{NEE_{CO2,uptake} }}{PAR}$ was relatively narrow (approximately 0.003–0.01) (Fig. 3). In contrast, in the low LUE zone (LUE < 0.002), the range of temperature and salinity were relatively low (~ ≤ 18 °C) and high (29–38 ppt), respectively. Although adequate sunlight was available for photosynthesis in the low LUE regime (average PAR = 1,130 µmol/m^2^/s)^45, 46^, the CO_2_ uptake (0.05–3 µmol/m^2^/s; Table 2) and corresponding LUE (< 0.002) were considerably low. Similar to the low LUE regime, salinity was very high (30–40 ppt) in the transitional regime where the range of temperature was comparable to the high LUE regime. The corresponding NEE_CO2,uptake_ and PAR were higher in the transitional regime than the low LUE regime, but lower than the high LUE regime. Atmospheric pressure, P_a_ varied from 1,008 to 1,019 millibar in the high LUE regime; the ranges of P_a_ in the transitional and low LUE regimes were also comparable to the high LUE regime (Table 2).

**Table 2:** Minimum (min), maximum (max) and mean of the daytime net uptake fluxes of CO_2_ and the associated environmental variables for the high, transitional, and low LUE $\left( {\frac{{NEE_{CO2,uptake} }}{PAR}} \right)$ regimes, as shown in Fig. 3.

Variable	High LUE regime			Transitional LUE regime			Low LUE regime		
	Min	Max	Mean	Min	Max	Mean	Min	Max	Mean
NEE_CO2,uptake_ (µmol/m^2^/s)	5	17	10	2	17	7	0.05	3	1
PAR (µmol/m^2^/s)	729	2,093	1,742	425	1,987	1,411	304	2,004	1,130
ST (°C)	17	26	21	16	26	20	9	18	14
SS (ppt)	10	30	25	30	40	33	29	38	33
P_a_ (millibar)	1,008	1,019	1,012	1,004	1,018	1,011	1,007	1,027	1,019

NEE_CO2,uptake_, PAR, ST, SS, and P_a_ refer, respectively, to daytime net uptake fluxes of CO_2_, photosynthetically active radiation, soil temperature, porewater salinity, and atmospheric pressure.

*LUE* refers to light use efficiency.

Figure 3 and Table 2 suggested two critical environmental thresholds (ST ~ 17 °C and SS ~ 30 ppt) that potentially defines the LUE regimes in coastal wetlands. Although the maximum temperature in the low LUE regime was 18 °C and minimum temperature in the transitional regime was 16 °C, we chose their intermediate temperature (ST ~ 17 °C) as the threshold, which also represented the minimum temperature of the high LUE regime. Overall, the salt marsh CO_2_ uptake followed the high regime when the temperature was approximately equal to or higher than 17 °C and salinity was lower or equal to 30 ppt (favorable condition for CO_2_ uptake). In contrast, the low LUE regime was subject to low temperature and high salinity (unfavorable condition for CO_2_ uptake) (Table 2). The transitional regime was mostly represented by high salinity stress (SS ≥ 30 ppt) with relatively favorable temperature (~ ≥ 16 °C) for photosynthesis—indicating a complex interplay between ST and SS in defining the transition from favorable uptake conditions to the unfavorable conditions.

## Discussion

The functional convergence of LUE with the BGC number indicates the emergent parametric reductions of net CO_2_ uptake fluxes in the *Spartina sp.* dominated salt marshes of Waquoit Bay and adjacent estuaries. Among the many definitions, LUE is conventionally defined as the net primary productivity (NPP) relative to the absorbed PAR^42, 43^. However, instead of NPP to PAR ratio, we defined LUE as the ratio of NEE_CO2,uptake_ to PAR in this paper. NEE_CO2,uptake_ includes gross primary productivity, and autotrophic as well as heterotrophic respirations during daytime hours. There is not much research available on the LUE of wetland plants, particularly for salt marshes. Overall, C_4_ plants such as *Spartina sp*. generally exhibit high photosynthetic efficiency relative to the C_3_ species^47^. For example, Jiang et al.^48^ reported a higher light utilization by the *Spartina sp.* plants than the C_3_ plants (*Phragmites australis* and *Scirpus mariqueter*) in the Yangtze River estuary, China. The comparatively higher LUE of C_4_ plants stems from their ability to capture light for an extended growing period, higher uptake rates, and favorable physiology and biochemistry^49^. Previous studies indicated a substantial influence of climatic, physiological, and biogeochemical factors (e.g., temperature, hydrology, canopy structure) on LUE for different species across ecosystems. Our study of the salt marsh CO_2_ fluxes and the corresponding LUE corroborates the finding of existing literature that LUE is characteristically linked with the dynamics of the BGC number (Fig. 3).

The derived dimensionless BGC number represented the combined effect of temperature and salinity, and enveloped the effect within a narrow range (approximately 0.04–0.17). The impact of BGC group on the response LUE, which varied from approximately 0.000009 to 0.01, facilitated the identification of three underlying governing regimes (i.e., high, transitional, and low) and emergence of a characteristics process diagram of the salt marsh CO_2_ fluxes. The identified soil temperature of ~ 17 °C and salinity of ~ 30 ppt thresholds dictated the transition of the CO_2_ uptake efficiency from high to low regimes. However, such limiting thresholds were not apparent in case of P_a_, although NEE_CO2,uptake_ can potentially decrease with increasing atmospheric pressure^39^. Therefore, we hypothesized that variations of ST and SS were the major factors responsible for regime transition of CO_2_ fluxes.

**Figure 4:**
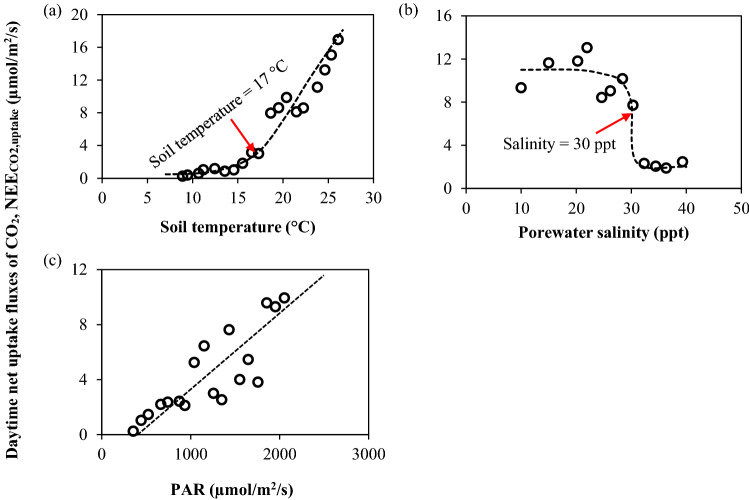
Plot of (**a**) soil temperature, (**b**) porewater salinity and (**c**) PAR with the corresponding NEE_CO2,uptake_ across the study marshes. The ST, SS, and PAR data were binned, respectively, for each 1 °C, 2 ppt, and 100 µmol/m^2^/s interval by pooling all the data across the four marshes. The bin averages of ST, SS, PAR and the corresponding average of NEE_CO2,uptake_ were then used to prepare the plot. The dotted line indicates the hypothetical trend line of NEE_CO2,uptake_ as a respective function of soil temperature, PAR or salinity. NEE_CO2,uptake_ refers to the daytime net uptake fluxes of CO_2_. PAR refers to the photosynthetically active radiation.

The identified 17 °C soil temperature threshold corresponds to the air temperature (AT) of 25.6 °C, which was obtained by developing a linear regression model of AT as a function of ST ($AT = 6.31 + 1.135 \times ST; R^{2} = 0.61$) using the measured data from the four salt marshes. The estimated threshold of AT = 25.6 °C was comparable to the limiting air temperature threshold (20–25 °C) for photosynthesis^10^. Sage and Kubien^10^ also reported the role of photosynthesis enzyme, RuBisCo as a limiting factor on the CO_2_ fixation of C_4_ plants in a comparatively cool environment (i.e., AT < 20 °C). The turnover rate of RuBisCo generally increases with temperature during daytime hours, and the rate corresponds to the release of RuBP (ribulose-1, 5-bisphosphate) that facilitates the CO_2_ fixation^10, 50^. Therefore, the low temperature in low LUE regime could represent the activation state of RuBisCo, where photosynthesis is extremely limited although the range of PAR in this regime was high enough to initiate a photosynthetic response (Table 2). The increase in CO_2_ uptake with the soil temperature of 17 °C or higher was further apparent in Fig. 4a, where a jump in CO_2_ uptake was observed at ST ~ 17 °C.

The most dynamic CO_2_ uptake characteristic was observed in the transitional regime, which was separated from the high LUE regime based on the salinity threshold of 30 ppt. Because of the very high salinity (SS ≥ 30 ppt), the uptake efficiency of the marshes decreased in the transitional regime, although the range of temperature in the transitional regime was comparable to the high LUE regime (Table 2). Previous studies reported a reduction of CO_2_ assimilation rate in *Spartina sp.* marshes when salinity was higher than 30 ppt^45, 51, 52^. High salinity significantly reduces the productivity by impacting the leaf chlorophyll content, protein synthesis, and lipid metabolism^11, 53, 54^. Further, high salinity-driven osmotic stress could expedite the stomatal conductance, which substantially hinders the photosynthesis rate^55^.

The observed reduction of CO_2_ uptake above 30 ppt salinity was further revealed in Fig. 4b, which showed a reverse S-shaped curve of CO_2_ fluxes in response to the changes in salinity from 10 to 40 ppt. The plot indicated an abrupt drop in NEE_CO2,uptake_ when salinity reached 30 ppt; further, NEE_CO2,uptake_ remained consistently low above the 30 ppt threshold. Therefore, we conjecture that temperature acts as a limiting factor to control the salt marsh CO_2_ uptake in the low LUE regime when ST is approximately equal to or less than ~ 17 °C. However, once temperature crosses the limiting threshold and reaches a favorable condition, CO_2_ uptake efficiency increases while salinity ~ 30 ppt emerges as a new limiting factor in controlling the LUE. It is worth mentioning here another limiting salinity threshold of 18 ppt above which the net CO_2_ uptake in tidal marshes is remarkably greater than the methane (CH_4_) emissions^56^. Overall, the high LUE regime corresponds to the “most favorable” climatic-biogeochemical condition (high temperature and low salinity) of CO_2_ uptake, whereas the low LUE regime corresponds to the “least favorable” condition (low temperature and high salinity) of CO_2_ uptake. In contrast, the transitional regime represents relatively favorable climatic condition (relatively high temperature) but unfavorable biogeochemical condition (high salinity) for CO_2_ uptake.

The CO_2_ fixation efficiency in *Spartina sp.* marshes can also be low because of light saturation^45^. However, NEE_CO2,uptake_ was observed to have a linear increasing trend with PAR (Fig. 4c) in the salt marshes at Cape Cod, indicating no clear impacts of photo-inhibition or light saturation on the productivity during our sampling periods in May–October of 2013. Further, any reduction of LUE due to a lack of water availability (hydraulic limitation) was unlikely to occur for plants in these salt marshes, since the wetland soil remained mostly saturated due to the tidal inundations^7, 57^.

The developed process diagram and insights into the governing regimes provide crucial information on the productivity and biogeochemical sensitivity of the coastal wetlands. Whether the salt marshes are efficiently assimilating CO_2_ under ambient environmental conditions can be determined from Fig. 3. Salt marshes that consistently follow the low LUE regime would likely require intervention to make them more efficient. For example, any upward shift of a marsh from the low to transitional regime could potentially be achieved by reducing the salinity (i.e., decreasing BGC) with the enhancement of the freshwater inflow. However, ongoing wetland restoration practices involve augmentation of the tidal flow, which increases the salinity of the system and in turn inhibits CH_4_ emission^56, 58^. Therefore, our study highlights a need for a robust optimization in tidal flow control and salinity to make a balance between plants’ productivity and CH_4_ emissions for an efficient system. Further, as high CO_2_ uptake rate reduces the radiative forcing and contribute to global cooling^59^, the identified regimes and characteristic diagram may be leveraged to map the potential coastal hotspots for radiative cooling.

Based on the process diagram (Fig. 3), the wetland managers can determine the operating regimes and rank the target wetlands in order of LUE. Although instantaneous fluxes and associated environmental variables were used to develop the diagram, the seasonal averages of the variables can be used to compute the corresponding BGC and LUE values. For example, by using the site-specific average values (May–October) of NEE_CO2,uptake_ and the major drivers, the corresponding representative LUE and BGC numbers for the salt marshes were computed (Table 3). By plugging in the computed site-specific BGC and LUE values in Fig. 3, it was found that on average Sage Lot Pond, Great Pond and Eel Pond had been operating under the transitional LUE regime during the 2013 summer period. In contrast, the Hamblin Pond overall represented the high LUE regime. Comparatively lower mean salinity in the Hamblin Pond than the other ponds (Table 3, Figure S2) made the pond more efficient in CO_2_ uptake relative to the available light. The results render these marshes strong candidates to be included in carbon crediting program as they are operating under transitional to high LUE regimes.

**Table 3:** Operating LUE regimes of the four study salt marshes using site-specific May–October, 2013 average values of NEE_CO2,uptake_ and corresponding major drivers.

Study salt marshes	Sage lot pond	Hamblin pond	Great pond	Eel pond
NEE_CO2,uptake_ (µmol/m^2^/s)	5.5	6.9	4.5	4.1
PAR (µmol/m^2^/s)	1,511.7	1,602.5	1,065.1	1,211.5
ST (°C)	17.5	18.7	17.2	17.1
SS (ppt)	31.0	26.0	31.0	34.0
P_a_ (millibar)	1,015.4	1,011.3	1,016.6	1,018.8
LUE (unitless)	0.004	0.004	0.004	0.003
BGC (unitless)	0.131	0.111	0.131	0.143
Operating LUE regime	Transitional	High	Transitional	Transitional

The units of the variables were converted based on the conversion factors showed in Table 2 to compute the corresponding LUE and BGC numbers. The operating regimes for each of the ponds were determined by plugging in the computed BGC and LUE values in the developed characteristics diagram shown in Fig. 3.

NEE_CO2,uptake_, PAR, ST, SS, and P_a_ refer, respectively, to daytime net uptake fluxes of CO_2_, photosynthetically active radiation, soil temperature, porewater salinity, and atmospheric pressure. LUE refers to the dimensionless light use efficiency number $\left( {\frac{{NEE_{CO2,uptake} }}{PAR}} \right)$ and BGC refers to dimensionless biogeochemical number $\left( {\frac{{ST \cdot c_{p} \cdot SS}}{{P_{a} }}} \right)$. ppt refers to parts per thousand. The value of constant specific heat of wet soil, c_p_ = 1.48 J/g/K.

The analysis was conducted by using a single extended growing season data from four *Spartina sp.* dominated coastal salt marshes, although they represented a gradient in nitrogen loading in Waquoit Bay and adjacent estuaries. Data from other coastal wetlands across environmental and plant community gradients should be incorporated in future research to test the generalizability of the process diagram and critical thresholds for the net uptake fluxes of CO_2_. Further, the presented parametric reductions and dimensional analysis method could be employed to develop similar process diagrams for other greenhouse gas (GHG) fluxes (e.g., CH_4_). However, application of similitude and dimensional analysis requires a proper understanding of the underlying mechanisms and processes. Successful outcomes from the Buckingham pi theorem largely depend on an accurate selection of major processes and relevant variables. Involving too few or too many variables in the analysis would lead to misleading results. Further, the most meaningful outcomes (i.e., dimensionless groups) demand an exploration of all possible combinations of repeating and non-repeating variables in the analysis. However, once an appropriate and mechanistically meaningful set of dimensionless numbers such as LUE and BGC are identified through parametric reductions, these can indicate different environmental (lowly, transitional, and highly efficient) regimes of the targeted GHG fluxes. For example, the process diagram of CO_2_ fluxes provided an overall understanding of the ecological health and carbon storage potential of the salt marshes in Cape Cod, MA. The information could be leveraged to spatially map coastal wetlands with a lower LUE (than 0.002; see Fig. 3) and higher BGC (than 0.13) to layout a detailed, but targeted sampling plan for restoration to a lower BGC and higher LUE. The research can, therefore, aid an efficient monitoring and management of coastal wetland carbon in a changing environment.

## Conclusions

The study successfully tested the hypothesis that the net uptake fluxes of CO_2_ in coastal salt marshes follow emergent ecological parameter reductions (similitudes) and distinct environmental regimes by using dimensional analysis. The research provided new insights into the first principles of wetland CO_2_ processes. Two meaningful and important dimensionless numbers (“light use efficiency” and “biogeochemical”) were identified based on an application of the Buckingham pi theorem. A semi-logarithmic plot of the dimensionless numbers demonstrated the emergence of a process diagram, which was characterized by three governing light use efficiency (LUE) regimes (high, transitional, and low). Subject to the appropriate temperature and salinity conditions (soil temperature ≥ 17 °C and salinity ≤ 30 ppt) suitable for photosynthesis, the high LUE regime favors the most efficient utilization of available sunlight by wetland plants. In contrast, the CO_2_ uptake rate relative to light was very low in the low LUE regime because of unfavorable ambient environmental conditions. Based on the characteristic process diagram, two critical environmental thresholds were identified (soil temperature ~ 17 °C and salinity ~ 30 ppt) to indicate regime transitions and wetland productivity.

The presented characteristic process diagram and environmental regimes would aid a targeted and efficient monitoring and management of tidal wetlands based on their CO_2_ uptake efficiency. The temperature and salinity thresholds render valuable knowledge of salt marsh productivity and resiliency in response to changes in key environmental drivers. The study also highlights the importance of developing environmental regime-specific predictive models of CO_2_ fluxes, compared to wetland-specific models, to minimize the site-specific model calibrations and uncertainty. The study can be considered a pioneering step in characterizing similitude-based emergent patterns and in identifying important process regimes of CO_2_ and other GHG fluxes in coastal salt marshes.

## Supplementary information

Supplementary Information.

## Data Availability

Data used in this study are described in main text, figures, tables, and Supplemental notes. The complete dataset is available in the figshare data repository at https://doi.org/10.6084/m9.figshare.9856439. The dataset is also available at https://sites.google.com/view/ecological-water/data-and-models.
