# Supplementary material for: Ecological parameter reductions, environmental regimes, and characteristic process diagram of carbon dioxide fluxes in coastal salt marshes
Source: Sci Rep. 2020 Sep 25;10:15732. doi: 10.1038/s41598-020-72066-8 (PMC7519661; doi:10.1038/s41598-020-72066-8)
Supplement: Supplementary file 1 — Supplementary Information. [file 41598_2020_72066_MOESM1_ESM.pdf]

Ecological parameter reductions, environmental regimes, and characteristic process diagram of carbon dioxide fluxes in coastal salt marshes

Khandker S. Ishtiaq[1] and Omar I. Abdul-Aziz[1 †]

[1]West Virginia University, PO BOX 6103, Morgantown, WV 26506-6103, U.S.A.

[†]Corresponding author; e-mail: oiabdulaziz@mail.wvu.edu; phone: +1 304 293 9929; fax: +1 304 293 7109.

Supplemental Notes

Table S1: Data summary (sample size = 137) for CO_2 fluxes and the associated environmental variables collected during May-October 2013 at the four salt marshes in Waquoit Bay and adjacent estuaries, MA.

Variable	Units	Mean	Standard deviation	Minimum	Maximum
$NEE_{CO2,uptake}$	µmol/m^2/s	5.33	4.72	0.05	17.10
PAR	µmol/m^2/s	1395.53	519.73	303.7	2093.08
ST	^{o}C	17.57	4.15	8.89	26.10
SS	ppt	30.50	4.70	10.00	40.00
P_a	millibar	1014.97	6.68	1004	1027

Note: $NEE_{CO2,uptake}$, PAR, ST, SS, and P_a refer, respectively, to the daytime net uptake fluxes of CO_2, photosynthetically active radiation, soil temperature, porewater salinity, and atmospheric pressure. ppt refers to parts per thousand.

Table S2: The full set of dimensionless pi (Π) numbers derived from various iterations.

*I	Repeating variables	Remaining variables	Derived dimensionless numbers ($\Pi_1, \Pi_2, \Pi_3, \Pi_4, \Pi_5$)
1	PAR, ST, SS, t	$NEE_{CO2,uptake}, P_a, c_p$	$\frac{NEE_{CO2,uptake}}{PAR}, \frac{SS \cdot P_a}{PAR^2}, \frac{ST \cdot c_p \cdot SS^2}{PAR^2}, \frac{ST \cdot c_p \cdot SS^3 \cdot P_a}{PAR^4}, \frac{ST \cdot c_p \cdot SS}{P_a}$
2	PAR, ST, P_a, t	$NEE_{CO2,uptake}, SS, c_p$	$\frac{NEE_{CO2,uptake}}{PAR}, \frac{SS \cdot P_a}{PAR^2}, \frac{PAR^2 \cdot ST \cdot c_p}{P_a^2}, \frac{ST \cdot c_p \cdot SS}{P_a}, \frac{SS \cdot P_a^3}{PAR^4 \cdot ST \cdot c_p}$
3	PAR, ST, c_p, t	$NEE_{CO2,uptake}, SS, P_a$	$\frac{NEE_{CO2,uptake}}{PAR}, \frac{SS \cdot \sqrt{ST \cdot c_p}}{PAR}, \frac{P_a}{PAR \cdot \sqrt{ST \cdot c_p}}, \frac{SS \cdot P_a}{PAR^2}, \frac{ST \cdot c_p \cdot SS}{P_a}$
4	PAR, SS, c_p, t	$NEE_{CO2,uptake}, ST, P_a$	$\frac{NEE_{CO2,uptake}}{PAR}, \frac{ST \cdot c_p \cdot SS^2}{PAR^2}, \frac{SS \cdot P_a}{PAR^2}, \frac{ST \cdot c_p \cdot SS^3 \cdot P_a}{PAR^4}, \frac{ST \cdot c_p \cdot SS}{P_a}$
5	PAR, P_a, c_p, t	$NEE_{CO2,uptake}, ST, SS$	$\frac{NEE_{CO2,uptake}}{PAR}, \frac{PAR^2 \cdot ST \cdot c_p}{P_a^2}, \frac{SS \cdot P_a}{PAR^2}, \frac{ST \cdot c_p \cdot SS}{P_a}, \frac{SS \cdot P_a^3}{PAR^4 \cdot ST \cdot c_p}$

*I refers to the iteration number with different sets of repeating variables

Table S3: Results of one-way ANOVA to compare the light use efficiency (LUE) values among high, transitional, and low LUE regimes across the four salt marshes.

Source of variation	Sum of squares	Degrees of freedom	Mean square	F-statistics	F-critical	p-value
			High vs. transitional LUE regimes			
Between regimes	0.0002	1	0.00018	32.70	3.93	< 0.0001
Within regimes	0.0006	103	0.00001			
Total	0.0008	104				
			High vs. low LUE regimes			
Between regimes	0.0006	1	0.00057	175.92	3.97	< 0.0001
Within regimes	0.0002	72	0.000003			
Total	0.0008	73				
			Transitional vs. low LUE regimes			
Between regimes	0.0002	1	0.00018	48.15	3.94	< 0.0001
Within regimes	0.0003	93	0.000004			
Total	0.0005	94				

Note: Significance of difference was evaluated at the 95% level of confidence (p-value < 0.05).

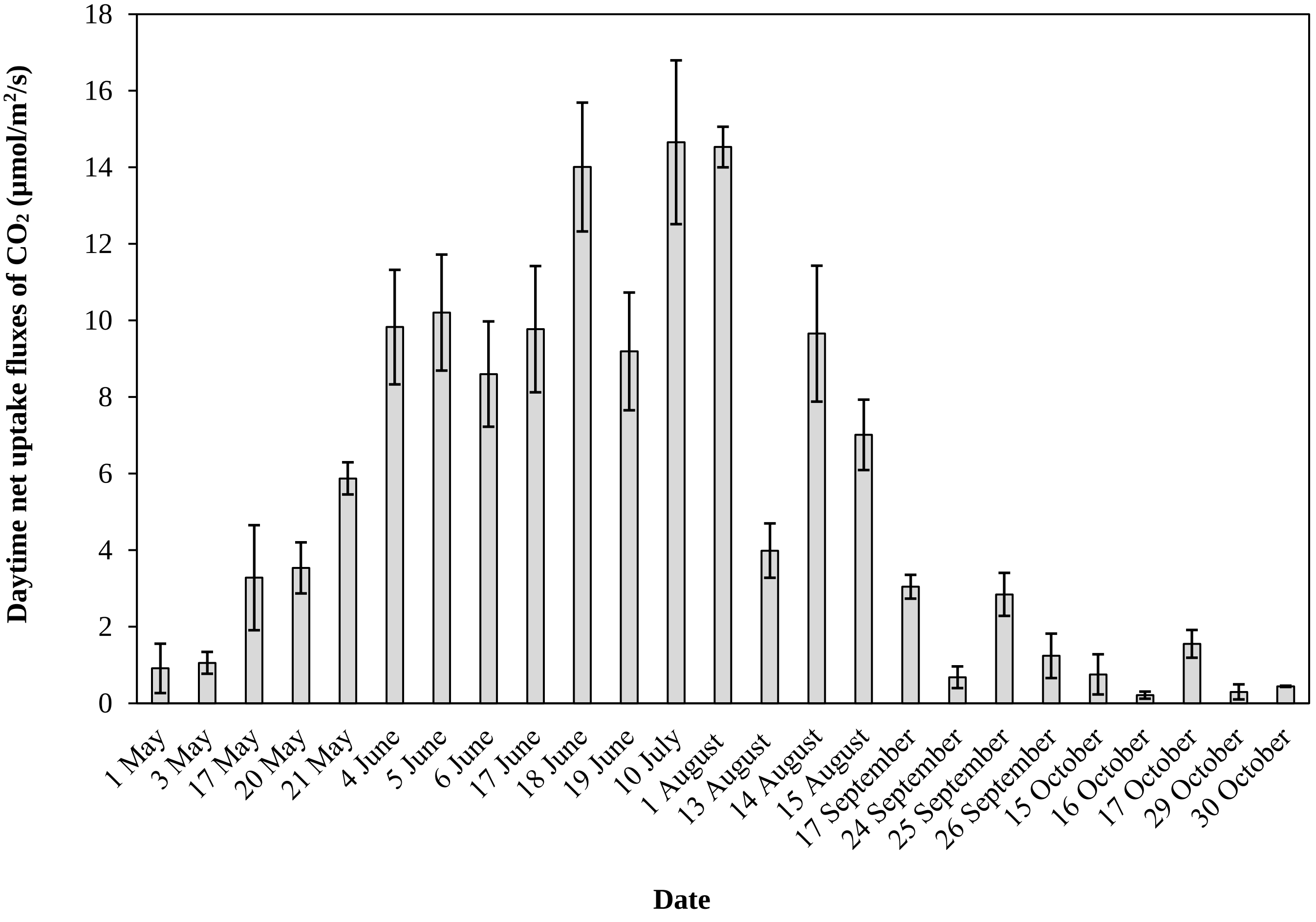

Figure S1: Average daytime net uptake fluxes of CO_2 measured in different growing season days during May-October 2013 across the four salt marshes in Waquoit Bay and adjacent estuaries, MA. Error bars indicate respective standard deviations of the measured fluxes on each date.

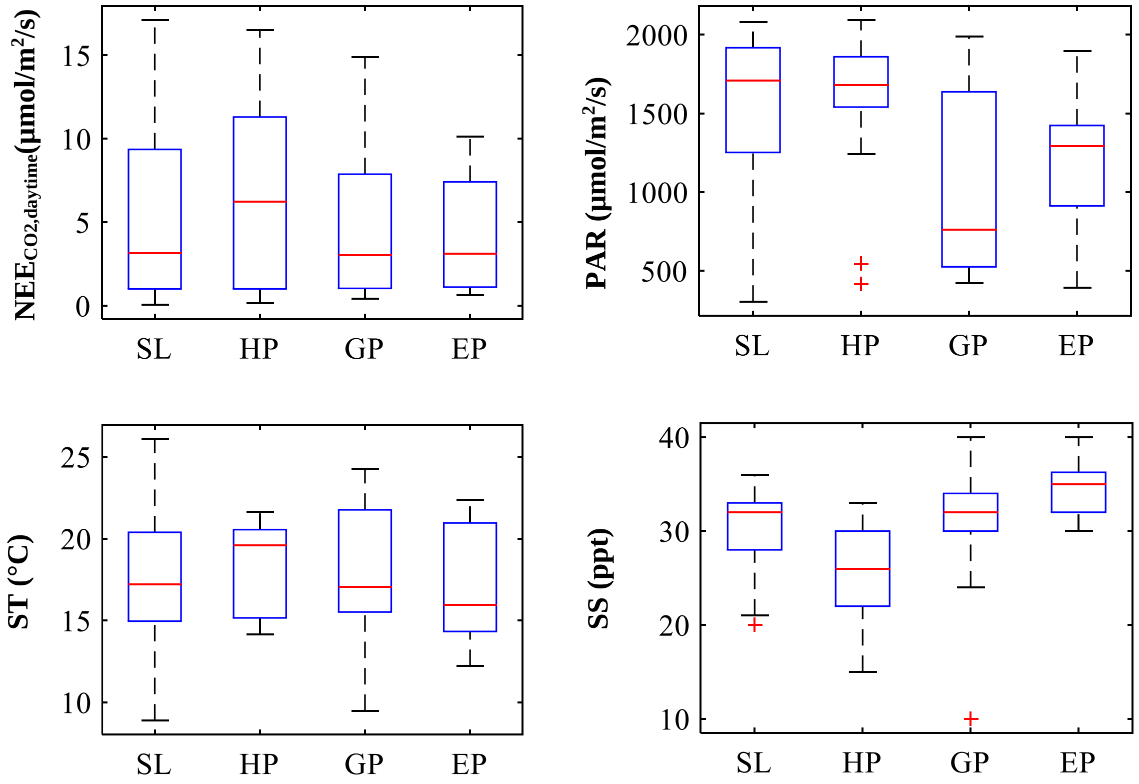

Figure S2: Boxplots showing the variability of the measured CO_2 fluxes and environmental variables for different salt marshes in Waquoit Bay and adjacent estuaries, MA. Here, $NEE_{CO2,uptake}$, PAR, ST, SS, refer, respectively, to the daytime net uptake fluxes of CO_2, photosynthetically active radiation, soil temperature, porewater salinity. SL, HP, GP, and EP refer to Sage Lot Pond, Hamblin Pond, Great Pond, and Eel Pond, respectively.

Text S1: Theoretical derivations of the dimensionless pi numbers using Buckingham pi theorem

Fundamental dimensions = 4 (M, L, T and K).

Total number of variables, $n = 7$;

Number of fundamental dimensions, $r = 4$;

The possible dimensionless (Π) numbers, $n - r = 7 - 4 = 3$.

$f = (NEE_{CO2,uptake}, PAR, ST, SS, P_a, c_p, t)$

$\Phi(\Pi_1, \Pi_2, \Pi_3) = 0$

<u>Iteration-1:</u>

Repeating variables = $PAR, ST, SS,$ and t

$\Pi_1 = (PAR)^a \cdot (ST)^b \cdot (SS)^c \cdot (t)^d \cdot NEE_{CO2,uptake}$

Based on the principle of dimensional homogeneity,

$M^0 \cdot L^0 \cdot T^0 \cdot K^0 = \left(\frac{M}{L^2T}\right)^{\mathrm{a}} \cdot (K)^b \cdot \left(\frac{M}{L^3}\right)^c \cdot (T)^d \cdot \frac{M}{L^2T}$

Therefore, $M^0 \cdot L^0 \cdot T^0 \cdot K^0 = M^{\mathrm{a+c+1}} \cdot L^{-2\mathrm{a}-3c-2} \cdot T^{-a+d-1} \cdot K^b$

Equating the exponents of M, L, T, and K on both sides yields,

$a + c + 1 = 0$

$-2a - 3c - 2 = 0$

$-a + d - 1 = 0$

$b = 0$

Solving using the method of substitutions, we get

$\mathrm{a} = -1, b = 0, c = 0, d = 0;$

Therefore, $\Pi_1 = \frac{NEE_{CO2,uptake}}{PAR}$

$\Pi_2 = (PAR)^a \cdot (ST)^b \cdot (SS)^c \cdot (t)^d \cdot P_a$

Based on the principle of dimensional homogeneity,

$$M^0 \cdot L^0 \cdot T^0 \cdot K^0 = \left(\frac{M}{L^2T}\right)^a \cdot (K)^b \cdot \left(\frac{M}{L^3}\right)^c \cdot (T)^d \cdot \frac{M}{LT^2}$$

Therefore, $M^0 \cdot L^0 \cdot T^0 \cdot K^0 = M^{a+c+1} \cdot L^{-2a-3c-1} \cdot T^{-a+d-2} \cdot K^b$

Equating the exponents of M, L, T, and K on both sides yields,

$a + c + 1 = 0$

$-2a - 3c - 1 = 0$

$-a + d - 2 = 0$

$b = 0$

Solving using the method of substitutions, we get

$a = -2, b = 0, c = 1, d = 0;$

Therefore, $\Pi_2 = \frac{SS \cdot P_a}{PAR^2}$

$\Pi_3 = (PAR)^a \cdot (ST)^b \cdot (SS)^c \cdot (t)^d \cdot c_p$

Based on the principle of dimensional homogeneity,

$$M^0 \cdot L^0 \cdot T^0 \cdot K^0 = \left(\frac{M}{L^2T}\right)^a \cdot (K)^b \cdot \left(\frac{M}{L^3}\right)^c \cdot (T)^d \cdot \frac{L^2}{T^2K}$$

Therefore, $M^0 \cdot L^0 \cdot T^0 \cdot K^0 = M^{a+c} \cdot L^{-2a-3c+2} \cdot T^{-a+d-2} \cdot K^{b-1}$

Equating the exponents of M, L, T, and K on both sides yields,

$a + c = 0$

$-2a - 3c + 2 = 0$

$-a + d - 2 = 0$

$b - 1 = 0$

Solving using the method of substitutions, we get

$a = -2, b = 1, c = 2, d = 0;$

Therefore, $\Pi_3 = \frac{ST \cdot c_p \cdot SS^2}{PAR^2}$

Furthermore,

$$\Pi_4 = \Pi_2 \times \Pi_3 = \frac{ST \cdot c_p \cdot SS^3 \cdot P_a}{PAR^4}$$

$$\Pi_5 = \frac{\Pi_3}{\Pi_2} = \frac{ST \cdot c_p \cdot SS}{P_a}$$

Therefore, the functional relationship between the response and predictor Π numbers can be expressed as follows:

$$\frac{NEE_{CO2,uptake}}{PAR} = \varphi\left[\left(\frac{SS \cdot P_a}{PAR^2}\right),\left(\frac{ST \cdot c_p \cdot SS^2}{PAR^2}\right),\left(\frac{ST \cdot c_p \cdot SS^3 \cdot P_a}{PAR^4}\right),\left(\frac{ST \cdot c_p \cdot SS}{P_a}\right)\right]$$

Iteration-2:

Repeating variables = $PAR, ST, P_a, and\ t$

$$\Pi_1 = (PAR)^a \cdot (ST)^b \cdot (P_a)^c \cdot (t)^d \cdot NEE_{CO2,uptake}$$

Based on the principle of dimensional homogeneity,

$$M^0 \cdot L^0 \cdot T^0 \cdot K^0 = \left(\frac{M}{L^2T}\right)^a \cdot (K)^b \cdot \left(\frac{M}{LT^2}\right)^c \cdot (T)^d \cdot \frac{M}{L^2T}$$

Therefore, $M^0 \cdot L^0 \cdot T^0 \cdot K^0 = M^{a+c+1} \cdot L^{-2a-c-2} \cdot T^{-a-2c+d-1} \cdot K^b$

Equating the exponents of M, L, T, and K on both sides yields,

$a + c + 1 = 0$

$-2a - c - 2 = 0$

$-a - 2c + d - 1 = 0$

$b = 0$

Solving using the method of substitutions, we get

$a = -1, b = 0, c = 0, d = 0;$

Therefore, $\Pi_1 = \frac{NEE_{CO2,uptake}}{PAR}$

$$\Pi_2 = (PAR)^a \cdot (ST)^b \cdot (P_a)^c \cdot (t)^d \cdot SS$$

Based on the principle of dimensional homogeneity,

$$M^0 \cdot L^0 \cdot T^0 \cdot K^0 = \left(\frac{M}{L^2T}\right)^a \cdot (K)^b \cdot \left(\frac{M}{LT^2}\right)^c \cdot (T)^d \cdot \frac{M}{L^3}$$

Therefore, $M^0 \cdot L^0 \cdot T^0 \cdot K^0 = M^{a+c+1} \cdot L^{-2a-c-3} \cdot T^{-a-2c+d} \cdot K^b$

Equating the exponents of M, L, T, and K on both sides yields,

$a + c + 1 = 0$

$-2a - c - 3 = 0$

$-a - 2c + d = 0$

$b = 0$

Solving using the method of substitutions, we get

$a = -2, b = 0, c = 1, d = 0;$

Therefore, $\Pi_2 = \frac{SS \cdot P_a}{PAR^2}$

$\Pi_3 = (PAR)^a \cdot (ST)^b \cdot (P_a)^c \cdot (t)^d \cdot c_p$

Based on the principle of dimensional homogeneity,

$$M^0 \cdot L^0 \cdot T^0 \cdot K^0 = \left(\frac{M}{L^2 T}\right)^a \cdot (K)^b \cdot \left(\frac{M}{LT^2}\right)^c \cdot (T)^d \cdot \frac{L^2}{T^2 K}$$

Therefore, $M^0 \cdot L^0 \cdot T^0 \cdot K^0 = M^{a+c} \cdot L^{-2a-c+2} \cdot T^{-a-2c+d-2} \cdot K^{b-1}$

Equating the exponents of M, L, T, and K on both sides yields,

$a + c = 0$

$-2a - c + 2 = 0$

$-a - 2c + d - 2 = 0$

$b - 1 = 0$

Solving using the method of substitutions, we get

$a = 2, b = 1, c = -2, d = 0;$

Therefore, $\Pi_3 = \frac{PAR^2 \cdot ST \cdot c_p}{P_a^2}$

Furthermore,

$$\Pi_4 = \Pi_2 \times \Pi_3 = \frac{ST \cdot c_p \cdot SS}{P_a}$$

$$\Pi_5 = \frac{\Pi_2}{\Pi_3} = \frac{SS \cdot P_a^3}{PAR^4 \cdot ST \cdot c_p}$$

Therefore, the functional relationship between the response and predictor Π numbers can be expressed as follows:

$$\frac{NEE_{CO2,uptake}}{PAR} = \varphi\left[\left(\frac{SS \cdot P_a}{PAR^2}\right), \left(\frac{PAR^2 \cdot ST \cdot c_p}{P_a^2}\right), \left(\frac{ST \cdot c_p \cdot SS}{P_a}\right), \left(\frac{SSP_a^3}{PAR^4 \cdot ST \cdot c_p}\right)\right]$$

Iteration-3:

Repeating variables = $PAR, ST, c_p, and\ t$

$\Pi_1 = (PAR)^a \cdot (ST)^b \cdot (c_p)^c \cdot (t)^d \cdot NEE_{CO2,uptake}$

Based on the principle of dimensional homogeneity,

$M^0 \cdot L^0 \cdot T^0 \cdot K^0 = \left(\frac{M}{L^2T}\right)^a \cdot (K)^b \cdot \left(\frac{L^2}{T^2K}\right)^c \cdot (T)^d \cdot \frac{M}{L^2T}$

Therefore, $M^0 \cdot L^0 \cdot T^0 \cdot K^0 = M^{a+1} \cdot L^{-2a+2c-2} \cdot T^{-a-2c+d-1} \cdot K^{b-c}$

Equating the exponents of M, L, T, and K on both sides yields,

$a + 1 = 0$

$-2a + 2c - 2 = 0$

$-a - 2c + d - 1 = 0$

$b - c = 0$

Solving using the method of substitutions, we get

$a = -1, b = 0, c = 0, d = 0;$

Therefore, $\Pi_1 = \frac{NEE_{CO2,uptake}}{PAR}$

$\Pi_2 = (PAR)^a \cdot (ST)^b \cdot (c_p)^c \cdot (t)^d \cdot SS$

Based on the principle of dimensional homogeneity,

$M^0 \cdot L^0 \cdot T^0 \cdot K^0 = \left(\frac{M}{L^2T}\right)^a \cdot (K)^b \cdot \left(\frac{L^2}{T^2K}\right)^c \cdot (T)^d \cdot \frac{M}{L^3}$

Therefore, $M^0 \cdot L^0 \cdot T^0 \cdot K^0 = M^{a+1} \cdot L^{-2a+2c-3} \cdot T^{-a-2c+d} \cdot K^{b-c}$

Equating the exponents of M, L, T, and K on both sides yields,

$a + 1 = 0$

$-2a + 2c - 3 = 0$

$-a - 2c + d = 0$

$b - c = 0$

Solving using the method of substitutions, we get

$a = -1, b = \frac{1}{2}, c = \frac{1}{2}, d = 0;$

Therefore, $\Pi_2 = \frac{SS \cdot \sqrt{ST \cdot c_p}}{PAR}$

$\Pi_3 = (PAR)^a \cdot (ST)^b \cdot (c_p)^c \cdot (t)^d \cdot P_a$

Based on the principle of dimensional homogeneity,

$M^0 \cdot L^0 \cdot T^0 \cdot K^0 = \left(\frac{M}{L^2T}\right)^a \cdot (K)^b \cdot \left(\frac{L^2}{T^2K}\right)^c \cdot (T)^d \cdot \frac{M}{LT^2}$

Therefore, $M^0 \cdot L^0 \cdot T^0 \cdot K^0 = M^{a+1} \cdot L^{-2a+2c-1} \cdot T^{-a-2c+d-2} \cdot K^{b-c}$

Equating the exponents of M, L, T, and K on both sides yields,

$a + 1 = 0$

$-2a + 2c - 1 = 0$

$-a - 2c + d - 2 = 0$

$b - c = 0$

Solving using the method of substitutions, we get

$a = -1, b = -\frac{1}{2}, c = -\frac{1}{2}, d = 0;$

Therefore, $\Pi_3 = \frac{P_a}{PAR \cdot \sqrt{ST \cdot c_p}}$

Furthermore,

$$\Pi_4 = \Pi_2 \times \Pi_3 = \frac{SS \cdot P_a}{PAR^2}$$

$$\Pi_5 = \frac{\Pi_2}{\Pi_3} = \frac{ST \cdot c_p \cdot SS}{P_a}$$

Therefore, the functional relationship between the response and predictor Π numbers can be expressed as follows:

$$\frac{NEE_{CO2,uptake}}{PAR} = \varphi\left[\left(\frac{SS \cdot \sqrt{ST \cdot c_p}}{PAR}\right), \left(\frac{P_a}{PAR \cdot \sqrt{ST \cdot c_p}}\right), \left(\frac{SS \cdot P_a}{PAR^2}\right), \left(\frac{ST \cdot c_p \cdot SS}{P_a}\right)\right]$$

Iteration-4:

Repeating variables = $PAR, SS, c_p,$ and t

$$\Pi_1 = (PAR)^{\mathrm{a}} \cdot (SS)^b \cdot (c_p)^c \cdot (t)^d \cdot NEE_{CO2,uptake}$$

Based on the principle of dimensional homogeneity,

$$M^0 \cdot L^0 \cdot T^0 \cdot K^0 = \left(\frac{M}{L^2T}\right)^{\mathrm{a}} \cdot \left(\frac{M}{L^3}\right)^b \cdot \left(\frac{L^2}{T^2K}\right)^c \cdot (T)^d \cdot \frac{M}{L^2T}$$

Therefore, $M^0 \cdot L^0 \cdot T^0 \cdot K^0 = M^{\mathrm{a+b+1}} \cdot L^{-2\mathrm{a}-3b+2c-2} \cdot T^{-a-2c+d-1} \cdot K^{-c}$

Equating the exponents of M, L, T, and K on both sides yields,

$a + b + 1 = 0$

$-2a - 3b + 2c - 2 = 0$

$-a - 2c + d - 1 = 0$

$-c = 0$

Solving using the method of substitutions, we get

$a = -1, b = 0, c = 0, d = 0;$

Therefore, $\Pi_1 = \frac{NEE_{CO2,uptake}}{PAR}$

$$\Pi_2 = (PAR)^{\mathrm{a}} \cdot (SS)^b \cdot (c_p)^c \cdot (t)^d \cdot ST$$

Based on the principle of dimensional homogeneity,

$$M^0 \cdot L^0 \cdot T^0 \cdot K^0 = \left(\frac{M}{L^2T}\right)^{\mathrm{a}} \cdot \left(\frac{M}{L^3}\right)^b \cdot \left(\frac{L^2}{T^2K}\right)^c \cdot (T)^d \cdot K$$

Therefore, $M^0 \cdot L^0 \cdot T^0 \cdot K^0 = M^{\mathrm{a+b}} \cdot L^{-2\mathrm{a}-3b+2c} \cdot T^{-a-2c+d} \cdot K^{-c+1}$

Equating the exponents of M, L, T, and K on both sides yields,

$a + b = 0$

$-2a - 3b + 2c = 0$

$-a - 2c + d = 0$

$-c + 1 = 0$

Solving using the method of substitutions, we get

$a = -2, b = 2, c = 1, d = 0;$

Therefore, $\Pi_2 = \frac{ST \cdot c_p \cdot SS^2}{PAR^2}$

$\Pi_3 = (PAR)^{\mathrm{a}} \cdot (SS)^b \cdot (c_p)^c \cdot (t)^d \cdot P_a$

Based on the principle of dimensional homogeneity,

$$M^0 \cdot L^0 \cdot T^0 \cdot K^0 = \left(\frac{M}{L^2T}\right)^{\mathrm{a}} \cdot \left(\frac{M}{L^3}\right)^b \cdot \left(\frac{L^2}{T^2K}\right)^c \cdot (T)^d \cdot \frac{M}{LT^2}$$

Therefore, $M^0 \cdot L^0 \cdot T^0 \cdot K^0 = M^{\mathrm{a+b+1}} \cdot L^{-2\mathrm{a}-3b+2c-1} \cdot T^{-a-2c+d-2} \cdot K^{-c}$

Equating the exponents of M, L, T, and K on both sides yields,

$a + b + 1 = 0$

$-2a - 3b + 2c - 1 = 0$

$-a - 2c + d - 2 = 0$

$-c = 0$

Solving using the method of substitutions, we get

$a = -2, b = 1, c = 0, d = 0;$

Therefore, $\Pi_3 = \frac{SS \cdot P_a}{PAR^2}$

Furthermore,

$$\Pi_4 = \Pi_2 \times \Pi_3 = \frac{ST \cdot c_p \cdot SS^3 \cdot P_a}{PAR^4}$$

$$\Pi_5 = \frac{\Pi_2}{\Pi_3} = \frac{ST \cdot c_p \cdot SS}{P_a}$$

Therefore, the functional relationship between the response and predictor Π numbers can be expressed as follows:

$$\frac{NEE_{CO2,uptake}}{PAR} = \varphi\left[\left(\frac{ST \cdot c_p \cdot SS^2}{PAR^2}\right), \left(\frac{SS \cdot P_a}{PAR^2}\right), \left(\frac{ST \cdot c_p \cdot SS^3 \cdot P_a}{PAR^4}\right), \left(\frac{ST \cdot c_p \cdot SS}{P_a}\right)\right]$$

Iteration-5:

Repeating variables = $PAR, P_a, c_p,$ and t

$\Pi_1 = (PAR)^{\mathrm{a}} \cdot (P_a)^b \cdot (c_p)^c \cdot (t)^d \cdot NEE_{CO2,uptake}$

Based on the principle of dimensional homogeneity,

$$M^0 \cdot L^0 \cdot T^0 \cdot K^0 = \left(\frac{M}{L^2T}\right)^{\mathrm{a}} \cdot \left(\frac{M}{LT^2}\right)^b \cdot \left(\frac{L^2}{T^2K}\right)^c \cdot (T)^d \cdot \frac{M}{L^2T}$$

Therefore, $M^0 \cdot L^0 \cdot T^0 \cdot K^0 = M^{\mathrm{a+b+1}} \cdot L^{-2\mathrm{a}-b+2c-2} \cdot T^{-a-2b-2c+d-1} \cdot K^{-c}$

Equating the exponents of M, L, T, and K on both sides yields,

$a + b + 1 = 0$

$-2a - b + 2c - 2 = 0$

$-a - 2b - 2c + d - 1 = 0$

$-c = 0$

Solving using the method of substitutions, we get

$a = -1, b = 0, c = 0, d = 0;$

Therefore, $\Pi_1 = \frac{NEE_{CO2,uptake}}{PAR}$

$$\Pi_2 = (PAR)^{\mathrm{a}} \cdot (P_a)^b \cdot (c_p)^c \cdot (t)^d \cdot ST$$

Based on the principle of dimensional homogeneity,

$$M^0 \cdot L^0 \cdot T^0 \cdot K^0 = \left(\frac{M}{L^2T}\right)^{\mathrm{a}} \cdot \left(\frac{M}{LT^2}\right)^b \cdot \left(\frac{L^2}{T^2K}\right)^c \cdot (T)^d \cdot K$$

Therefore, $M^0 \cdot L^0 \cdot T^0 \cdot K^0 = M^{\mathrm{a+b}} \cdot L^{-2\mathrm{a}-b+2c} \cdot T^{-a-2b-2c+d} \cdot K^{-c+1}$

Equating the exponents of M, L, T, and K on both sides yields,

$a + b = 0$

$-2a - b + 2c = 0$

$-a - 2b - 2c + d = 0$

$-c + 1 = 0$

Solving using the method of substitutions, we get

$a = 2, b = -2, c = 1, d = 0;$

Therefore, $\Pi_2 = \frac{PAR^2 \cdot ST \cdot c_p}{P_a^2}$

$$\Pi_3 = (PAR)^{\mathrm{a}} \cdot (P_a)^b \cdot (c_p)^c \cdot (t)^d \cdot SS$$

Based on the principle of dimensional homogeneity,

$$M^0 \cdot L^0 \cdot T^0 \cdot K^0 = \left(\frac{M}{L^2T}\right)^{\mathrm{a}} \cdot \left(\frac{M}{LT^2}\right)^b \cdot \left(\frac{L^2}{T^2K}\right)^c \cdot (T)^d \cdot \frac{M}{L^3}$$

Therefore, $M^0 \cdot L^0 \cdot T^0 \cdot K^0 = M^{\mathrm{a+b+1}} \cdot L^{-2\mathrm{a}-b+2c-3} \cdot T^{-a-2b-2c+d} \cdot K^{-c}$

Equating the exponents of M, L, T, and K on both sides yields,

$a + b + 1 = 0$

$-2a - b + 2c - 3 = 0$

$-a - 2b - 2c + d = 0$

$-c = 0$

Solving using the method of substitutions, we get

$a = -2, b = 1, c = 0, d = 0;$

Therefore, $\Pi_3 = \frac{SS \cdot P_a}{PAR^2}$

Furthermore,

$$\Pi_4 = \Pi_2 \times \Pi_3 = \frac{ST \cdot c_p \cdot SS}{P_a}$$

$$\Pi_5 = \frac{\Pi_3}{\Pi_2} = \frac{SS \cdot P_a^3}{PAR^4 \cdot ST \cdot c_p}$$

Therefore, the functional relationship between the response and predictor Π numbers can be expressed as follows:

$$\frac{NEE_{CO2,uptake}}{PAR} = \varphi\left[\left(\frac{PAR^2 \cdot ST \cdot c_p}{P_a^2}\right), \left(\frac{SS \cdot P_a}{PAR^2}\right), \left(\frac{ST \cdot c_p \cdot SS}{P_a}\right), \left(\frac{SS \cdot P_a^3}{PAR^4 \cdot ST \cdot c_p}\right)\right]$$
